# Salaries of Poor-Law Medical Officers

**Published:** 1914-04-11

**Authors:** 

**Affiliations:** Hon. Sec. Poor-Law Medical Officers' Association


### THE EDITOR'S LETTER-BOX.

### Salaries of Poor-Law Medical Officers.

*To the Editor of* The Hospital.

Sir,—While I thoroughly endorse much of your criticism of the action of the Local Government Board in preventing progressive Boards of Guardians from adequately remunerating their medical officers, I must take exception to your eulogies of the Bermondsey scheme. You speak of it as a "well-thought-out scheme," and opinions may, no doubt, differ as to this; but you are perhaps unaware that the principle of it—viz., that the present system of independent district medical officers should be abolished and replaced by assistant medical officers acting under the medical superintendent of the infirmary—has been considered and condemned both by our Association and by the British Medical Association.

We consider it very necessary, in the interests of the sick poor, that the district medical officers should be independent, and have their tenure of office protected by the Medical Appointments Order of 1857. You have perhaps not observed that one of the objections of the Bermondsey Guardians to the conditions of the Local Government Board is that the latter desire the assistant medical officers, who are to take the place of district medical officers, to have security of tenure for five years; but the Guardians wish them to be entirely in their hands, so as to be able to dismiss them at any time. In appointments of this kind it is not only the gross amount of salary that has to be considered, but the permanence of the office also. Many Poor-Law medical officers would prefer security of tenure with a moderate salary to the same office at a much higher salary, but with no security against arbitrary dismissal.—I am, yours truly,

Major Greenwood,
Hon. Sec. Poor-Law Medical
Officers' Association.

[Dr. Greenwood's criticism of the proposals of the Bermondsey Guardians to reorganise their system of medical relief, from the point of view of the existing district medical officers, is cogent so far that a new scheme, such as the one under discussion, applied to an old system, necessarily has the disadvantage of the displacing of officers who have done good work in the past. No such scheme should, therefore, be undertaken lightly. But the advantages of concentrating the whole work of medical relief in the case of a small and densely populated area like Bermondsey are of a nature that outweigh all personal considerations. The great defect of the system under which outdoor relief is carried on by a staff of part-time district medical officers and indoor relief by the staff of the infirmary lies in the complete dissociation of the indoor work from the outdoor work. When a patient is sent into the infirmary by a district medical officer the infirmary staff have no knowledge of the treatment he has been receiving nor of his condition before admission nor of the character of his home. Similarly, when the patient is discharged the infirmary staff lose touch with him and the district medical officer is ignorant of what has happened in the infirmary. Of course, these defects could be remedied by an interchange of information between the indoor and outdoor staffs, but in practice there is no time for such co-operation. A whole-time staff under one head allows the medical superintendent of the infirmary to have a general view of the whole medical requirements of the parish. The Bermondsey scheme, though it may inflict hardship in individual cases, has been arranged so as to reduce this hardship to a large extent. The existing district medical officers are to be invited to apply for the whole-time assistant medical officerships, and, if they apply, they are to be appointed. Failing this, they are to be superannuated in accordance with the Poor-Law Officers' Superannuation Act. The provision thus made for the old officers is certainly not a generous one, and it would be only fair under the special circumstances for the Guardians to add ten years' service to each officer displaced in computing the superannuation allowance. Dr. Greenwood seems to miss the point of the Local Government Board's desire to have the proposed new assistant medical officers appointed for a term of five years. The Guardians' proposal is to appoint them on the same terms as every other assistant medical officer of a Poor-Law infirmary, that is, for an unlimited period, subject to determination by the Board of Guardians without reference to the Local Government Board. The Local Government Board wishes the appointments to be made under the same conditions as to dismissal, but for five years only, and for the whole scheme to be reconsidered at the end of that period. The Guardians rightly object that such a definite limitation of the tenure of office would prevent many good candidates from applying. The interests of the sick poor are protected by the fact that the medical superintendent is not subject to dismissal by the Guardians except with the consent of the Local Government Board. It is in his hands, and not in that of the assistant medical officers, that the direction of the general lines of the medical work will lie. The responsibility rests upon him, and upon him alone, and he is protected from unjust dismissal by the terms of his appointment.—Ed. The Hospital.]

